# The polymerase milieu of human papillomavirus

**DOI:** 10.1128/jvi.01698-25

**Published:** 2026-08-03

**Authors:** Keira R. Ylvisaker, Gopishankar Thirumoorthy, Emma Wisniewski, Kavi P. M. Mehta

**Affiliations:** 1 Department of Comparative Biosciences, University of Wisconsin-Madison School of Veterinary Medicine 70738, Madison, Wisconsin, USA; Universiteit Gent, Merelbeke, Belgium

**Keywords:** HPV, tumor viruses, DNA damage, DNA repair, mutagenesis, replication, replisome

## Abstract

High-risk human papillomaviruses (HPVs) cause cervical, anogenital, and head and neck cancers. A key oncogenic mechanism involves viral exploitation of host DNA damage tolerance pathways to support productive replication, a process that also drives genomic instability. Despite evidence that specific cellular polymerases participate in HPV genome replication, the full complement of polymerases deployed across the viral life cycle remains poorly defined. This minireview examines the potential roles of both replicative and translesion synthesis (TLS) polymerases at distinct stages of the HPV life cycle, and new tools that can be used to assess their potential importance in driving cancer, the life cycle, and altering mutagenic potential.

## INTRODUCTION

Shope papillomavirus was first discovered in 1933 to cause benign, hyperproliferative lesions in cottontail rabbits that could be induced to undergo malignant conversion following treatment with genotoxic agents. This provided early and compelling evidence that viruses could contribute to carcinogenesis in concert. By the 1970s, epidemiological observations had established that cervical neoplasia behaved as a sexually transmitted disease. Herpes simplex virus type 2, cytomegalovirus, and papillomaviruses were each proposed as candidate etiological agents of cervical cancer (1). It was Harald zur Hausen who, beginning with the molecular cloning of HPV16 from cervical carcinoma biopsies, codified the central role of HPV in cervical carcinogenesis, work for which he was awarded the Nobel Prize. We now know that high-risk HPV types account for approximately 5% of the global cancer burden, including virtually all cervical cancers, most oropharyngeal cancers, and significant proportions of anal, vulvar, vaginal, and penile cancers (2). Although most infections are cleared, persistent infection with high-risk types drives malignant progression.

HPVs have circular, double-stranded DNA genomes that are approximately 8 kilobases in length and encode between six and eight open reading frames, along with splice variants, organized into three functional regions: the early region, the late region, and the upstream regulatory region (URR), also termed the long control region (LCR) (3). The URR harbors the origin of replication, E1- and E2-binding sites, and binding sites for host transcription factors. E1 is an ATP-dependent helicase that was discovered in BPV and, together with E2, assembles at the viral origin of replication within the URR to initiate bidirectional DNA replication (4, 5). E2 functions as both a transcriptional activator and repressor. E2 represses the promoter that drives expression of the E6 and E7 oncogenes, such that loss of E2, which frequently occurs upon viral genome integration into host DNA, leads to E6 and E7 overexpression (6–8). E2 also tethers the episomal viral genome to host mitotic chromosomes during cell division along with host factors such as SMC1, ensuring faithful partitioning to daughter cells (9–11). E4, expressed as a spliced E1^E4 fusion transcript, accumulates in the upper differentiating epithelial layers and disrupts the cytokeratin network, facilitating virion assembly and release (12, 13). E5 is a small, hydrophobic transmembrane protein that modulates host growth factor receptor signaling, notably altering epidermal growth factor receptor activity, and contributes to immune evasion (14–16).

E6 and E7 are the principal oncoproteins and are arguably the most studied HPV gene products; they are also often the only viral genes consistently retained and expressed in HPV-associated cancers. E6 recruits the E6AP ubiquitin ligase to target p53 for proteasomal degradation, abrogating cell cycle arrest and apoptotic responses (17, 18). E6 also activates telomerase through transcriptional upregulation of hTERT for immortalization (19–21). E7 binds and promotes the degradation of the retinoblastoma tumor suppressor protein (pRb) and related pocket proteins p107 and p130, liberating E2F transcription factors and driving unscheduled S-phase entry (22–25). Finally, the late region encodes L1 and L2, the major and minor capsid proteins, respectively. L1 can self-assemble into icosahedral virus-like particles. L2 facilitates genome encapsidation and viral entry.

**Fig 1:**
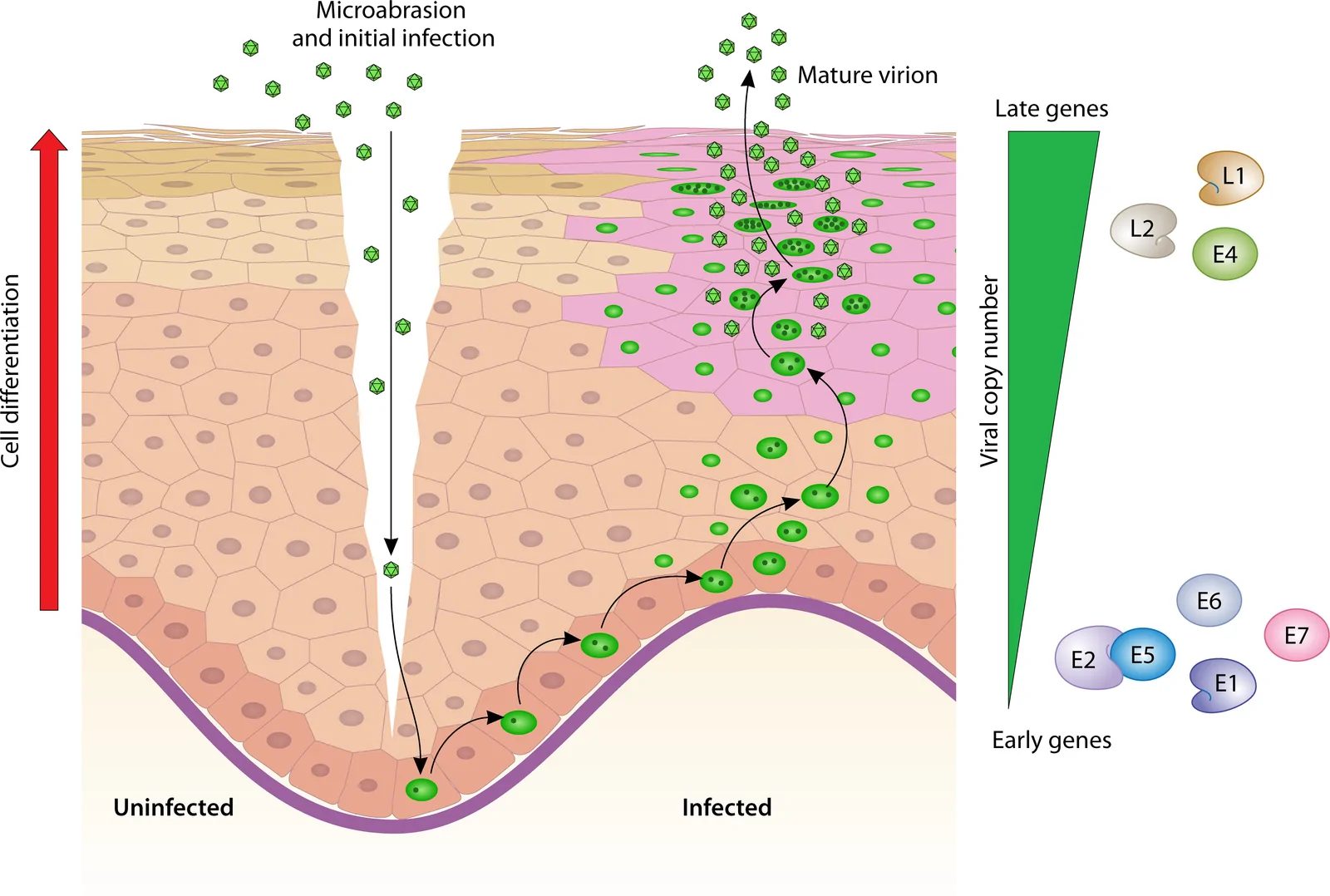
Schematic of HPV differentiation-dependent life cycle. Viral copy number increases upon differentiation, leading to a switch in modes of replication from theta-mediated replication to rolling-circle/recombination-associated replication.

The viral life cycle is intimately coupled to the squamous cell epithelial differentiation program (Fig. 1). PVs infect basal keratinocytes, accessing them through microabrasions, and establish their genome as a low-copy-number extrachromosomal element (episome) in a process known as establishment. In these undifferentiated basal cells, early gene expression is maintained at low levels, and the episomal copy number is maintained through a process known as *viral maintenance,* which occurs through theta-structure intermediates in normal S-phase (26). As infected daughter cells migrate suprabasally and initiate the terminal differentiation program, the viral life cycle is activated in a stepwise fashion: E6 and E7 maintain the differentiating cell in a replication-competent state, E1 and E2 drive amplification of the viral genome to high copy numbers, and this is thought to be by rolling circle or recombination-mediated replication (26–28). E1^E4 restructures the cellular architecture, and L1 and L2 are expressed exclusively in the uppermost differentiated layers to encapsidate newly replicated genomes (29). Mature virions are sloughed from the keratinous epithelial surface.

Despite encoding multiple proteins, papillomaviruses are fundamentally dependent on host factors for their productive life cycle. Unlike many DNA viruses, PVs do not encode their own DNA polymerases and instead co-opt host replication machinery. E1 helicase activity unwinds the viral origin, but DNA synthesis itself is carried out by host polymerases (5). Studies suggest that E1 directly interacts with DNA polymerase α-primase (Polα) to recruit it to the viral origin, initiating RNA-primed DNA synthesis on the viral template (30–32). Single-stranded binding protein RPA, Topoisomerase I, and Polε are similarly recruited to support processive replication fork progression (33, 34). E2 additionally serves as the assembly factor that loads E1 onto the origin (as originally determined in BPV) but also participates in recruiting host replication and repair factors to viral genomes (35, 36).

This dependence on host machinery counterintuitively extends to an intimate and exploitative relationship with the DNA damage response (DDR). The oncogenes and E1 and E2 induce genomic instability and double-strand breaks (37–42). Both viral infection and oncogene expression activate ATM- and ATR-dependent signaling cascades, and these responses are functionally required for efficient viral replication (37, 43, 44). Key DDR effectors, including but not limited to γ-H2AX, members of the MRN complex (MRE11-RAD50-NBS1), BRCA1, RAD51, TOPBP1, and the cohesin component SMC1 (recruited by viral-encoded CTCF sites), are recruited to discrete viral replication foci in differentiating cells (11, 36, 38, 40, 45–48). The activation of homologous recombination factors at viral replication centers is thought to support recombination-dependent replication while HPV E7 subverts NHEJ by dysregulating RNF168 (28, 42, 49). Additionally, studies suggest that HPV may sequester DNA replication and repair factors from the host DNA to its own viral genome, diluting their activity (40). E1-driven viral replication on the episome generates replication intermediates, including stalled forks, converging forks, and recombination-associated structures (concatemers), that are sensed and processed by the host DDR (28). Indeed, multiple studies indicate the presence of roadblocks on the HPV genome itself (discussed below). The virus hijacks DNA repair factors, diverting them from the host genome to viral DNA to facilitate rapid and faithful amplification, which has consequences. Host genomic DNA is left under-repaired, contributing to the accumulation of breaks and genomic instability that characterize HPV-associated cancers (38, 40). The dependence of the viral life cycle on an active DDR also creates a potential therapeutic vulnerability, as pharmacologic disruption of DDR signaling impairs viral replication and may selectively sensitize HPV-positive cells to DNA-damaging agents.

To date, no systematic experimental dissection across the complete viral life cycle has delineated the precise contributions of the high-fidelity polymerases, Polα, Polδ, and Polε, at each replicative stage. These assumptions additionally do not resolve the question of how these viruses manage replication under conditions of chronic replication stress, activated DNA damage signaling, structures in the viral episome, and a differentiation program that progressively shuts down the host replisome and actively regulates and potentiates the viral life cycle across types. This gap is the central subject of this review.

## POLYMERASE REPERTOIRE

To understand how papillomaviruses may replicate their genomes, it is first necessary to appreciate the full repertoire of DNA polymerases encoded by the human genome. Human cells express at least 15 distinct DNA polymerases, plus the specialized primase PRIMPOL that interestingly shares similarities to the herpes virus primase UL52 (50, 51). These enzymes partition broadly into three functional categories: replicative synthesis, TLS, and DNA damage tolerance (DDT) polymerases.

The replicative polymerases, Polα-primase, Polδ, and Polε, carry out the bulk of chromosomal DNA synthesis with fidelity enforced by 3′-to-5′ exonuclease proofreading domains (Polδ and Polε) or, in the case of Polα, by rapid handoff to Polδ and Polε (52). Polγ, Polθ, and Polν function in mitochondrial replication, microhomology-mediated end joining (MMEJ), and interstrand crosslink repair and HR, respectively (53–56). Polβ, Polλ, Polμ, and terminal deoxynucleotidyl transferase (TdT) operate primarily in base excision repair and nonhomologous end joining and V(D)J recombination (57). Other TLS polymerases, Polζ, Polη, Polι, Polκ, and REV1, lack proofreading activity and synthesize DNA with substantially lower fidelity, but possess open active sites that accommodate bulky or distorted template lesions that would normally stall replicative polymerases (58). Finally, PRIMPOL performs both primase and polymerase activities and *de novo* DNA repriming downstream from lesions at stalled replication forks and is regulated by the downstream regulator of ATR, CHK1 toward DDT (59).

The conventional model of papillomavirus replication invokes only high-fidelity polymerases, and direct biochemical evidence exists specifically for Polα and Polε, despite clear evidence that HPV replication proceeds through mechanistically distinct phases during maintenance and productive amplification (theta structures vs recombination-mediated/rolling-circle replication) (26, 27). The principal argument against involvement of error-prone polymerases is that PV genomes are thought to be relatively stable over evolutionary time. This may be true, but neither high-fidelity nor low-fidelity polymerases have been mapped across the viral life cycle, nor have the existing population of viral genomes been sequenced at high-depth or with single-molecule approaches. Additionally, few if any studies have systematically assessed whether HPV genomes replicated in differentiating epithelium are indeed homogeneous in sequence following amplification. The focus of this review is to address and query whether the high-fidelity-only assumption accounts for viral replication across the full complexity of the HPV life cycle, or whether polymerases from the repair and TLS categories may play an unexpected role. Whether viral replication proceeds with high- and/or low-fidelity polymerases remains a fundamental unresolved question.

## HPV AND LOW-FIDELITY POLYMERASES: STATUS QUO

Evidence that links HPV to the dysregulation of TLS synthesis primarily comes from the Wallace laboratory, which has demonstrated that HPV-associated cervical cancers frequently exhibit elevated expression of multiple TLS pathway genes (60, 61). Mechanistically, HPV16 E7 can activate TLS pathway expression in a manner that depends in part on pRb degradation, consistent with an E2F-driven replication-stress tolerance program but also suggesting inactivation of Polη (61).

In cutaneous epithelia, β-HPV 8E6 converges on impaired TLS by destabilizing p300 and attenuating ATM/ATR signaling, thereby preventing Polη accumulation and foci formation after UV damage (62). Consistent with broader repair-pathway reprogramming, HPV8 E6 promotes repair of double-strand breaks through PARP1-dependent, Polθ-mediated alternative end joining (63). Previous studies also indicate that Polη can participate in E1- and E2-mediated HPV replication, strengthening the potential for error-prone HPV replication (64).

Beyond polymerase regulation, work from multiple groups demonstrates that HPV genomes can replicate through multiple modes during the viral life cycle (26, 28, 42, 65–67). In addition to canonical bidirectional theta replication, HPV DNA synthesis can generate intermediates that are consistent with recombination-dependent replication. Interestingly, *some* viral DNA synthesis persists in the presence of aphidicolin, which blocks Polε, Polδ, and Polα, suggesting a fidelity-independent mode of replication during viral genome amplification and maintenance (68).

Collectively, these studies demonstrate that HPV oncoproteins substantially reprogram the host DNA damage tolerance and repair environment, and that error-prone DNA synthesis pathways are poised to operate in HPV-associated cells (69). However, whether low-fidelity polymerases directly participate in HPV genome replication remains unresolved, and the functional importance of TLS activity for the viral life cycle has not yet been directly tested.

## HPV REPLICATION INTERMEDIATES AND STRUCTURE

**Fig 2:**
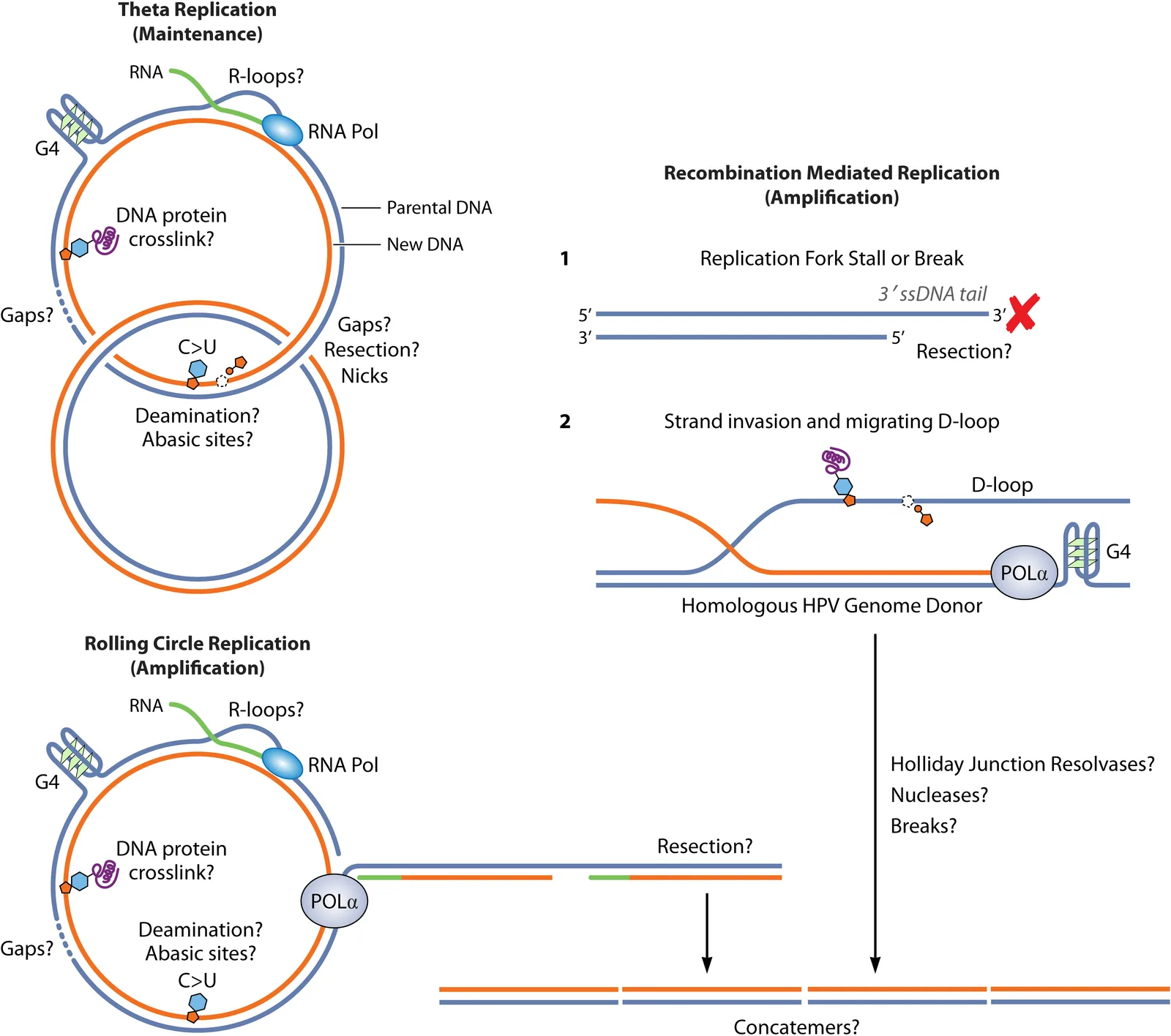
Curated schematic of potential HPV replication schemes and obstacles.

**Fig 3:**
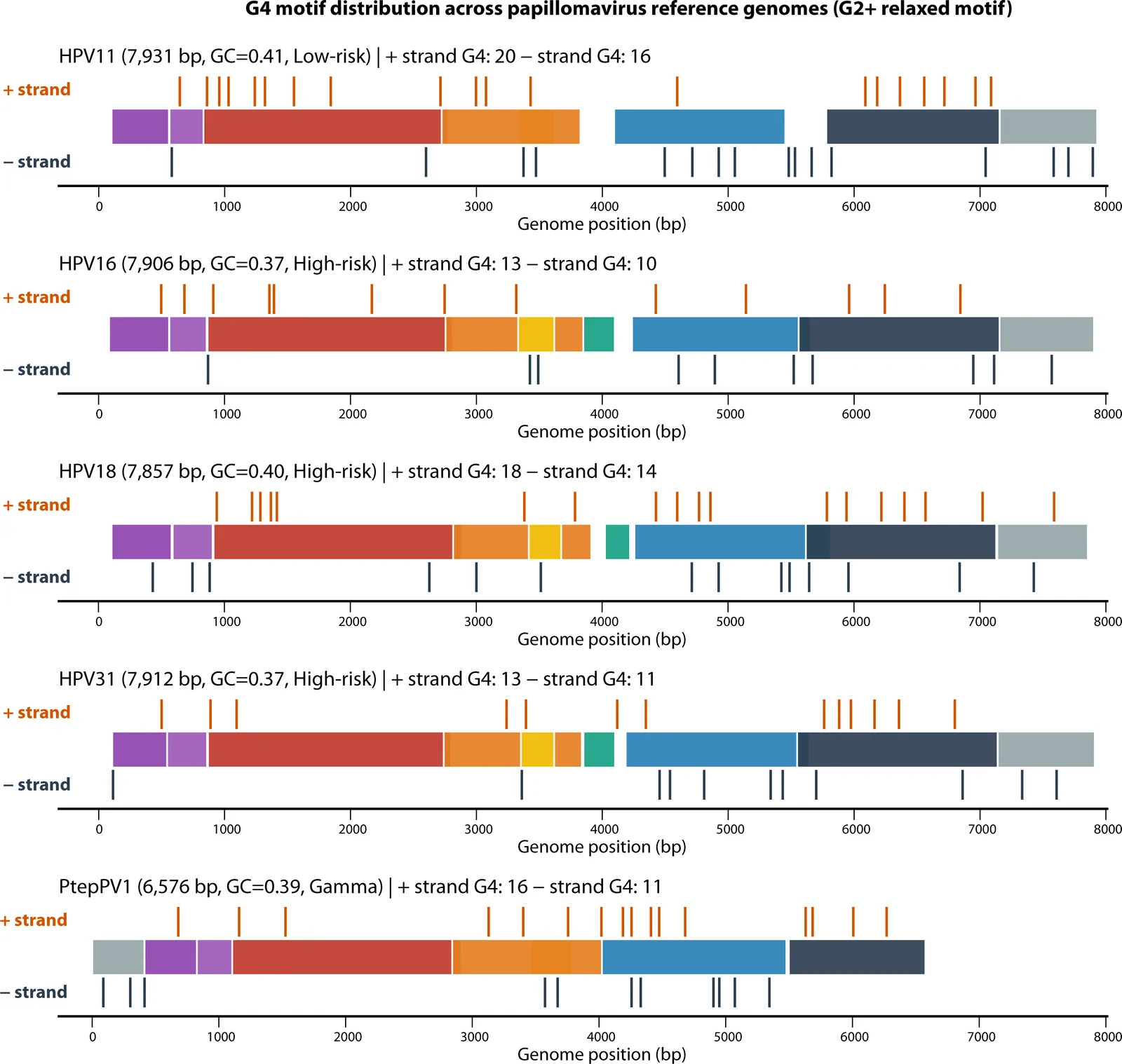
Computationally predicted putative G4 quadruplexes in high-risk and low-risk HPVs and in wild red colobus. The y-axis is count, and the x-axis is genome position. G4-forming sequences were predicted in each genome using G4Hunter (window 25 nt, score threshold 1.2), and observed counts were compared to a null distribution of 500 dinucleotide-preserving shuffles that control for base composition. All five genomes contained significantly more G4 loci than expected by chance (Z > 2.5, empirical *P* < 0.01); GC content indicated.

Studies suggest that HPV replication may generate multiple replication intermediates that require hijacking of host repair machinery to resolve (Fig. 2). In undifferentiated basal cells, the ~8 kb episome replicates via bidirectional theta structures from the URR-proximal origin through E1 and E2, with 2D gel analysis of HPV confirming double-Y and bubble intermediates consistent with converging fork theta replication (65). Upon keratinocyte differentiation, this shifts toward rolling circle synthesis, in an epithelial cell-specific, E1/E2-dependent manner, though more recent work argues the late-phase switch instead involves break-induced replication (BIR) and recombination-based replication, activated downstream of oncoprotein-driven replication stress in a variety of models (27, 67, 70). G4-forming sequences and R-loops add another layer of complexity: G-rich regions capable of forming stable G4s have been confirmed and are computationally predicted in the URR, L2, E1, and E4 regions of multiple HPV types, and these structures are even present in the recently discovered monkey gamma papillomavirus PtepV1 (Fig. 3) (71–74).

A single leading-strand G4 is sufficient to arrest the host CMG helicase by lodging within its central channel, generating fork uncoupling and ssDNA gaps that feed into downstream stress responses (75). R-loops formed by replication-transcription conflicts compound this idea of structural barrier formation in the HPV genome. R-loop levels are elevated in HPV-positive cells on both viral and cellular sequences, driven by E6-mediated p53 suppression, and their dysregulation directly impairs viral transcription and stable replication, while R-loop accumulation in precancerous HPV-positive cells is specifically associated with γ-H2AX and H3K36me3 at ATM, ATRX, RAD51C, and Fanconi Anemia (FA) pathway genes (40, 76, 77).

Downstream, HPV activates ATM to recruit HR factors including RPA, RAD51, and BRCA1 directly to viral replication foci, with this activity required specifically for differentiation-dependent amplification (38, 40, 45). TLS connects these nodes: TLS polymerases are activated by G4-induced ssDNA gaps under replication stress. REV1 is required for replication through G4 structures, and Polη and Polκ play complementary roles at and upstream of a G4 quadruplex (78, 79). G4s in the URR and E1 region of the HPV genome, stabilized by the supercoiled and transcriptionally active episome, would thus be predicted to engage TLS as a tolerance mechanism. It is important to note, however, that other evidence suggests that the RNF168-RAD18 axis, an upstream regulator of TLS, is dysregulated by HPV E7 (49).

Theta-mode replication sharpens two demands that are less acute on chromosomes. The episome carries a single origin with no dormant backup, so any stalled fork that cannot be repriming-rescued is lost. This puts PRIMPOL and the CHK1-PRIMPOL axis in a more essential position than in cellular replication. Bidirectional forks must also converge and terminate on a small circle, and the joint molecules formed at convergence require strand-displacement and bridging activities that Polθ provides. Replication-transcription conflicts on the heavily transcribed episome add a third pressure, with Polκ predicted at R-loop-associated stalls.

It is important to keep in mind that HPV amplification proceeds in differentiating keratinocytes under chronic DNA damage signaling and elevated oxidative stress. Additionally, the viral genome itself carries palindromes, AT/TA tracts, and G-quadruplex motifs that have been demonstrated to stall replicative polymerases. Pold and Pole may handle the bulk of synthesis, but these obstacles and the associated lesion load create specific contexts where bypass or repriming activity becomes necessary.

Under rolling circle amplification, the replisome needs processive leading-strand synthesis with strand displacement, plus a way to traverse the palindromes, AT-rich tracts, and potential G-quadruplexes in the early region and URR (80, 81). PRIMPOL is the natural candidate for repriming behind a displacement fork, with Polη and Polκ filling in across structured DNA or oxidative lesions on the displaced strand. The CHK1-PRIMPOL axis fits directly given ATR activation in the amplification compartment.

Under break-induced replication, the requirements could shift. POLD3-dependent migrating D-loop synthesis becomes the operating mode with the potential for Polζ and REV1 extending across lesions encountered during long-tract synthesis. Polθ could also resolve collapsed intermediates by microhomology-mediated synthesis, which also makes it a strong candidate at integration junctions.

Besides potential lesions, HPV replicative intermediates likely demand activities that Polδ and Polε do not robustly provide strand displacement at branched junctions, which could be engaged by Polθ, extension of RAD51-mediated D-loops (Polη or Polκ), and extension from the distorted primer termini generated by imperfect strand invasion (Polζ-REV1). This is a stronger case for TLS polymerase involvement than the obstacle-bypass argument, since intermediate processing is required at essentially every amplification event rather than only when a fork meets a lesion. Direct evidence on HPV episomes remains limited, and strand-specific mutational profiling of amplifying genomes is the most tractable test.

## SMALL DNA TUMOR VIRUSES AND HOST POLYMERASES

PVs are one of seven known viruses that can cause cancers in humans. These include KSHV, EBV, Merkel Cell Polyoma Virus, HTLV-1, Hepatitis B Virus, and Hepatitis C Virus. Of these, only HPV and MCPyV (polyoma) use host polymerases for replication. Polyomaviruses and papillomaviruses are both small nuclear circular double-stranded DNA tumor viruses, yet their replication environments suggest different relationships with host DNA polymerases. Polyomaviruses, including SV40, BK virus, JC virus, and Merkel cell polyomavirus, replicate using a mechanism that closely resembles canonical cellular DNA replication, and the SV40 origin of replication system has broadly informed the DNA replication field. The viral large T antigen binds the origin and functions as a helicase that recruits the host replisome; biochemical reconstitution experiments established that large T antigen together with 10 purified human proteins, including RPA, DNA polymerase α-primase, Polδ, PCNA, RFC, topoisomerases I and II, FEN1, and DNA ligase I, is sufficient to support complete, origin-dependent SV40 DNA replication *in vitro* (82–84). Absent from these factors is the major leading strand polymerase Polε, which has been shown to interact with the PV E1 helicase (34, 84). Interestingly, studies from the Morgan lab indicate that increased DNA damage signaling does not alter HPV E1-E2-mediated replication but does prevent SV40 large T-antigen-mediated replication, and DNA damage reduces the quality of E1-E2 replication (85, 86).

**Fig 4:**
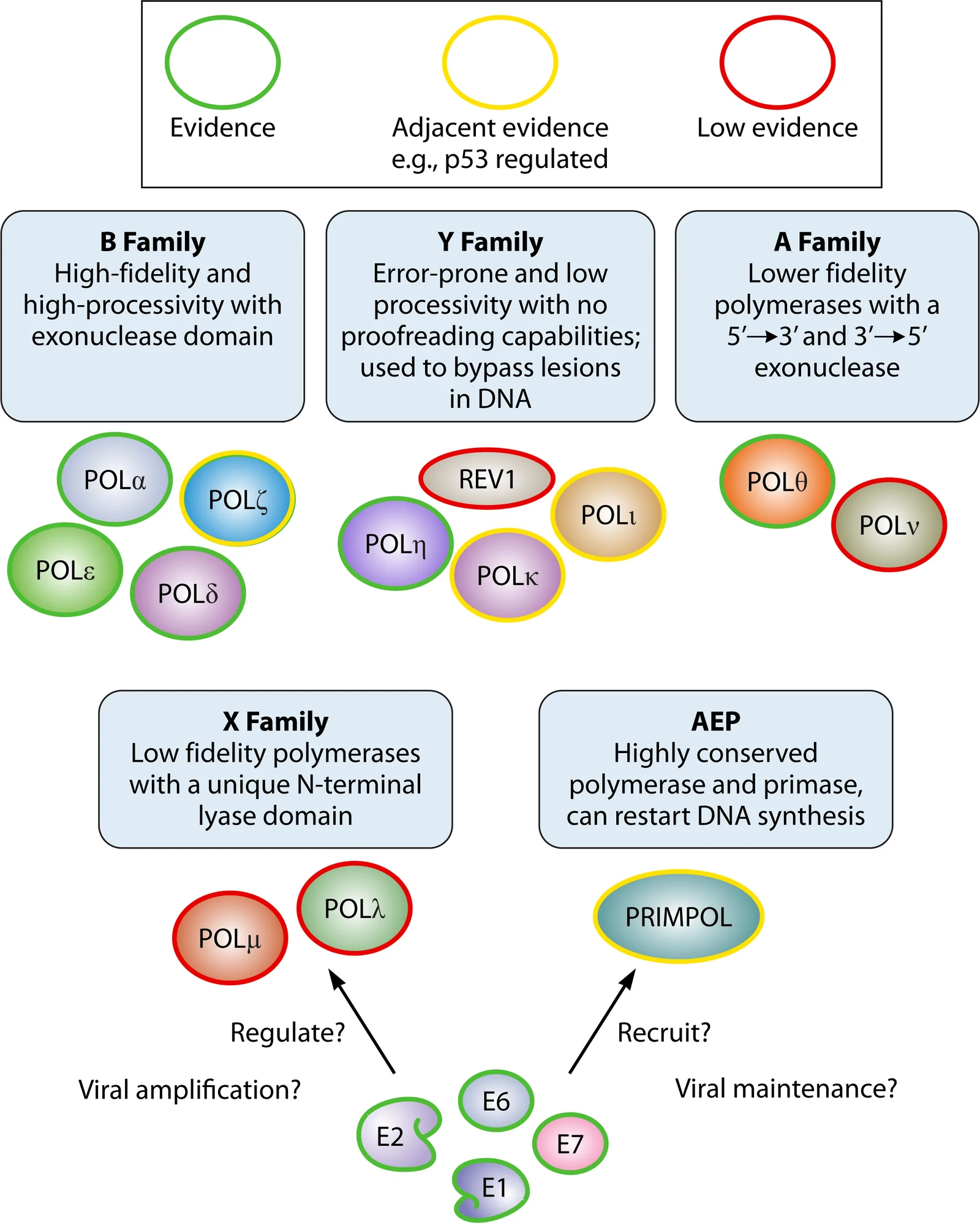
Curated schematic of the human polymerase milieu.

These observations suggest a broader evolutionary principle across small DNA tumor viruses. Polyomaviruses and PVs that encode helicases but not polymerases must replicate with host replication and repair pathways, making them sensitive and potentially dependent on DNA damage tolerance mechanisms such as TLS. In contrast, viruses that encode their own polymerases can maintain tighter control over replication fidelity and fork architecture. To understand how non-canonical polymerases such as TLS may be utilized in the PV polymerase milieu, it is important to understand each host polymerase (Fig. 4; Table 1). For the purposes of this review, we will not discuss Polγ, Polβ, or TdT.

**TABLE 1:** Evidence of polymerase involvement in HPV-associated infections in literature^*a*^

Polymerase	Family	Lesion type	HPV relevance
Polα	B	Abasic, thymine glycol	Replication initiation (30–32)
Polε	B	Mismatches	Replication (33, 34)
Polδ	B	Mismatches	Replication (33, 34)
Polθ	A	DSBs	Genome integration (87, 88)
Polη	Y	Thymine dimers, 6-4 photoproducts, G-quadruplexes	Direct relationship to p53 (60, 61)
REV1	Y	Abasic, N²/O^6^-alkylguanines, 6-4 photoproducts, G-quadruplexes	Chemoresistance (89), replication (90)
Polζ	B	8-oxos, thymine dimers, O^6^-alkylguanine, 6-4 photoproducts, 3-methyladenine	Chemoresistance (91)
Polκ	Y	Abasic, BPDE, thymine dimers, N^2^-alkylguanine, G-quadruplexes	Related to p53 regulation (92–94)
Polι	Y	Abasic, minor groove distortions	Related to p53 regulation (95)
Polμ	X	Small gaps	Episome stability (96)
PRIMPOL	AEP	8-oxos, 6-4 photoproducts	Related to CHK1 inhibition (97, 98)
Polλ	X	Abasic, 8-oxos, small gaps	Potential E6/E7 regulation point
Polν	A	Thymine glycol	Potential chemoresistance (99, 100)

^*a*^ Stronger evidence indicated by descending order.

## REV1 (*REV1,* REV1)

REV1 is an interesting member of the TLS family of proteins in that it is both important structurally as a scaffold for TLS activity but also has a weak polymerase activity. As a structural protein, it assembles the multi-polymerase TLS complex that can include Polη, Polι, Polκ, and Polζ to sites of damage (101). As a polymerase, it acts as a dCMP transferase that inserts cytosine opposite damaged bases like abasic sites that can be generated by APOBECs (102). REV1 responds to abasic sites and guanine adducts, including N²-alkylguanine lesions, and facilitates more error-free bypass of UV photoproducts in human cells (103, 104). It is appealing to consider its potential importance in HPV-infected cells and associated cancers, which overexpress APOBEC, a major driver of abasic sites in human cells (105, 106). Additionally, REV1 germline variants are associated with cervical cancer risk (107).

In cancer, REV1/Polζ-dependent TLS contributes to acquired chemoresistance (89). Cancer cells appear to rewire replication to favor TLS polymerases, suppressing ssDNA gaps that would otherwise limit fitness (108). Small-molecule REV1 inhibitors like JH-RE-06 sensitize ovarian cancer cells to cisplatin (89, 109).

REV1 is also important in virology. REV1, REV3L, and REV7 are all co-opted by HCMV for optimal replication, likely because the GC-rich, repeat-heavy herpesvirus genome accumulates lesions that the viral polymerase alone cannot handle, while Polη, Polκ, and Polι restrict viral replication (90). Intriguingly, in HSV-1, the same study found the opposite pattern: Polη, Polκ, and Polι were required for optimal replication, while depletion of Polζ/REV1 trended toward enhanced viral fitness (102).

## POLYMERASE ZETA (*REV3L/MAD2L2*, REV3L/REV7)

Polζ is unique among TLS polymerases for its ability to extend DNA synthesis past lesions, and its high processivity and fidelity place it within the B family of polymerases even though it lacks an exonuclease domain. Polζ has demonstrated the ability to bypass a variety of lesion types using different mechanisms. In BIR, where a DSB occurs, Polζ induces microhomology-mediated break-induced replication (MMBIR), which requires only a small amount of homologous DNA to succeed (110, 111). In yeast, Polζ bypasses thymine dimers and 6-4 photoproducts and can extend up to 200 base pairs past the lesion (112, 113). Interestingly, thymine dimers may be persistent in cells expressing HPV E6 (114). Polζ also bypasses distorted bases (e.g., bulky guanine adducts) (115).

In cervical cancer, levels of Polζ expression have been demonstrated as a marker for chemotherapy effectiveness, where elevated expression overall strongly correlated with resistance to chemoradiation in patients (116). REV7 has been shown to be significantly higher in cervical cancer tissue compared to non-cancerous tissue and is correlated with resistance to radiotherapy, although the mechanism employed is still unknown, along with REV3L (117). REV3L depletion sensitizes cervical cancer cells to cisplatin, whereas overexpression confers resistance (91).

## POLYMERASE KAPPA (*POLK*, Polκ)

Polκ, encoded by POLK, is a Y-family TLS polymerase specialized for replicating across bulky lesions that stall replicative polymerases (58, 92). It is the eukaryotic ortholog of bacterial DinB and exhibits a unique ability to bypass N^2^-alkylguanine adducts with relatively high fidelity (101, 118). Beyond classical TLS activity, Polκ also functions in replication fork stabilization and restart when encountering replication stress, positioning it as a critical factor in genome maintenance (79, 119).

Despite these insights, the role of Polκ in oncogenic viral contexts, particularly HPV infection, remains poorly characterized. Although TLS polymerases such as Polη have been implicated in HPV-associated dysregulation, direct evidence linking Polκ to viral replication or oncoprotein regulation remains limited.

Mechanistically, the transcriptional regulation of Polκ by p53 is context-dependent and bidirectional. Normally, wild-type p53 suppresses the Polκ promoter, and loss of p53 function correlates with elevated Polκ expression in human cancer tissues (93). However, in response to DNA damage, p53 can also drive Polκ upregulation through a damage-inducible transcriptional program. p21, acting downstream of p53, independently suppresses TLS polymerase engagement at PCNA. The net outcome of p53 loss on Polκ levels is due to the removal of basal promoter repression. In the context of HPV infection, E6-mediated degradation of p53 may similarly relieve this repression, providing a mechanistic rationale for Polκ upregulation in HPV-associated lesions. Whether this dysregulation occurs in HPV-infected cells remains to be directly demonstrated. This elevation may enhance the cell’s ability to tolerate replication-blocking lesions but also increases the risk of mutagenesis (92, 94).

Functionally, Polκ is recruited to stalled replication forks by PCNA ubiquitination and FA-associated factors to resume DNA synthesis following stalls and stress (79, 119). In the specific context of interstrand crosslink repair, FA pathway components also contribute to TLS polymerase coordination. FA pathways are linked to HPV-positive cells (99, 100, 120).

Polκ occupies a unique niche at the intersection of TLS, replication stress, and carcinogenesis. Although direct evidence for Polκ regulation by HPV remains to be demonstrated, the potential transcriptional repression via E6 and replication stress via E7 provides a mechanistic rationale for its involvement in HPV-associated mutagenesis and cancer progression. Future studies exploring Polκ activity in the context of viral oncoproteins may reveal novel targets for intervention in HPV-driven malignancies.

## POLYMERASE ETA (*POLH*, Polη)

As a member of the Y-family polymerases, Polη is known to bypass thymidine dimers commonly caused by UV damage with high fidelity and can also bypass 6-4 photoproducts but with lower fidelity. The large active site of Polη is unique among TLS polymerases and is likely the reason behind the accurate insertion of A-A adjacent to cis-syn thymidine dimers (121). Polη is regulated by p53, and ιτσ levels increase after UV exposure in a p53-dependent manner (122). Similarly to Polκ, Polη is recruited to lesions in DNA by protein REV1 and acts in parallel with PCNA to repair the replicating genome (123, 124).

Due to Polη’s ability to repair cyclobutene pyrimidine dimers (like T-T), loss or mutation in the POLH gene causes Xeroderma pigmentosum variant, where those with the condition are hypersensitive to cancers driven by UV damage like melanomas (125). Polη may also act as an oncogene in those undergoing chemotherapy, as the DNA damage being introduced to push cells into apoptotic pathways may be repaired by Polη, allowing cells to survive.

In HPV-positive systems, E6 and E7 alter Polη expression levels. HPV16 E6 impairs TLS at the effector level by degrading p53 and thereby blocking induction of Polη in response to replication stress (60, 61). Loss of Polη causes even more stress and eventually leads to fork collapse and carcinogenesis. Exogenous Polη restores TLS function and confers resistance to replication-stress–inducing agents including cisplatin, indicating that polymerase availability is rate-limiting. Polη may be important in the viral life cycle as early entry into the S-phase and chronic replication stress can require increased levels of TLS polymerases like Polη to achieve complete genome replication (126).

## POLYMERASE LAMBDA (*POLL*, Polλ)

Polλ is a unique polymerase that acts primarily in repair through BER and NHEJ and secondarily functions as a TLS polymerase. Polλ can bypass small gaps through its binding activity on both 3′ and 5′ ends, 8-oxoguanines with high fidelity, and abasic sites (127–130). Like other members of the X-family, Polλ has moderate fidelity across all mentioned lesion types and can complete faithful insertion in up to a 9 bp gap during BER (131).

In HPV-infected cells, high levels of oxidative damage are present, with 8-oxo-2′-guanosine products being left in its wake (132). Although no direct correlation has been uncovered between Polλ and HPV, this increase in the ideal substrate for Polλ indicates a unique possible point of regulation for HPV E6/E7.

## POLYMERASE IOTA (*POLI*, Polι)

Polι, another member of the Y-family polymerases, has been previously characterized as an extremely error-prone TLS polymerase with a high error rate and can substitute for Polη (133). Polι preferentially inserts deoxynucleotides opposing abasic sites or otherwise distorted regions of DNA in the minor groove, with an incidence of following Hoogsteen base-pairing (134, 135). Like Polκ and η, Polι has been delineated as an “inserter” polymerase but can extend synthesis based on a template of purine dNTPs.

Polι is a relatively understudied polymerase with no literature describing the relationship between it and HPV or other papillomaviruses. As previously described, HPV E6 recruits E6AP to degrade p53. p53 and Polι action may be correlated and may act together to promote fork reversal (more error-free repair) after DNA damage by chemical crosslinkers (136). Since p53 synthesis is high under cellular stress, some low levels of the tumor suppressor will persist even with proteasomal targeting by E6AP, which allows the p53-Polι construct to potentially increase DNA synthesis through TLS action instead of replication fork reversal (95). It was previously shown that hypoxia-inducible factor 1α (HIF-1α) is a major regulatory factor of Polι, and HPV E7 is a known enhancer for HIF-1α (137, 138). This suggests that E7 may modulate Polι activity through HIF-1α.

## POLYMERASE THETA (*POLQ*, Polθ)

Polθ is an unusual low-fidelity polymerase best known for its role in MMEJ, where it extends minimally paired DNA ends to repair double-strand breaks (139, 140). Unlike replicative polymerases, Polθ tolerates mismatches and damaged templates, often generating templated insertions and deletions that leave a distinctive mutational footprint in human genomes (141, 142).

In cancer, Polθ is frequently overexpressed and is associated with poor prognosis across multiple tumor types. Tumors with homologous recombination defects, such as BRCA1/2-deficient cancers, become highly dependent on Polθ-mediated repair for survival, creating a synthetic-lethal vulnerability that is now being therapeutically targeted (54, 55, 143).

In cervical cancer, Polθ has been identified as a gene associated with tumor progression, and its knockdown inhibits proliferation and migration in cervical cancer cell lines (144). Polθ is also among the most mutated DNA damage response genes in cervical cancers (145). Because HPV oncogenes induce chronic replication stress and DNA damage signaling, it is appealing to consider that Polθ may help repair broken or stalled replication intermediates generated during viral replication or integration. Although direct recruitment of Polθ by papillomaviruses has not yet been demonstrated, its ability to synthesize across damaged DNA and minimal homology suggests it could provide a flexible repair polymerase during viral genome maintenance. Two recent studies suggest an importance for MMEJ and Polθ-mediated MMEJ for viral integration, with E6 driving Polθ expression (87, 88).

## PRIMPOL (*PRIMPOL,* PRIMPOL)

PRIMPOL possesses both primase and polymerase activities (51, 146). Its defining function is the ability to *de novo* reprime DNA synthesis downstream of replication-blocking lesions or stalled fork structures, providing a dedicated restart mechanism that allows the replisome to bypass obstacles without requiring fork reversal or template switching (147). PRIMPOL synthesizes short DNA primers using its primase activity and can also perform limited TLS across certain lesions, including UV-induced 6-4 photoproducts and oxidative damage such as 8-oxoguanine, though with relatively low fidelity and processivity (148–150). PRIMPOL is recruited to stalled forks through its interaction with RPA-coated single-stranded DNA, placing it at precisely the structures that accumulate during replication stress, and is activated by CHK1 and PLK1 (59, 151, 152). Unlike classical TLS polymerases that operate through the RAD18-PCNA ubiquitination pathway, PRIMPOL acts through a mechanistically independent repriming route, making it a parallel rather than redundant tolerance pathway; however, remaining gaps are thought to be filled post-replicatively by other TLS polymerases.

In cancer, PRIMPOL’s role is emerging as an important factor. Tumor cells experiencing high levels of oncogene-driven replication stress may become dependent on PRIMPOL-mediated repriming to maintain viable fork progression and avoid lethal fork collapse (153). PRIMPOL can promote replication fork restart in BRCA1-deficient cells, where fork protection is already compromised, suggesting that it may serve as a compensatory survival mechanism in HR-defective tumors (154). Conversely, PRIMPOL-mediated repriming generates ssDNA gaps behind the fork, and the accumulation of these gaps has been linked to genome instability, chemosensitivity, and PARP inhibitor response (155).

In cervical cancer, PRIMPOL has not been directly investigated, but HPV-driven tumorigenesis provides a compelling rationale for its involvement. HPV E6 and E7 oncoproteins enforce constitutive S-phase entry and deregulate origin firing, generating chronic replication stress and CHK1 activation. CHK1 inhibition reduces HPV copy number and viral amplification, while CHK1 levels are higher in HPV-positive head and neck cancers (97, 98). During productive infection, the viral episome replicates using host machinery, and impediments such as secondary structures or transcription-replication collisions may require repriming to complete synthesis. The ssDNA gaps left by PRIMPOL could contribute to the genomic instability of advanced cervical cancers, and particularly the structural rearrangements at HPV integration sites, as persistent gaps are prone to breakage. Given that ssDNA gap accumulation is a critical determinant of PARP inhibitor and platinum sensitivity and that cervical cancers show PARP inhibitor responsiveness, understanding PRIMPOL’s status in these tumors may have direct therapeutic relevance.

## POLYMERASE MU (*POLM,* Polμ) AND NU (*POLN,* Polη)

DNA polymerases μ (Polμ) and ν (Polν) are two relatively undercharacterized repair polymerases that contribute to genome maintenance through distinct but complementary mechanisms. Polμ is closely related to terminal deoxynucleotidyl transferase (TdT) and is best known for its importance in non-homologous end joining (NHEJ), where it fills short gaps at broken DNA ends to facilitate ligation (156). Uniquely among gap-filling polymerases, Polμ can perform both template-dependent and minimally templated nucleotide additions, enabling it to bridge DNA ends that share little or no sequence complementarity (157–159). Polμ interacts with the Ku70/80–XRCC4–Ligase IV complex, positioning it directly at double-strand break sites during classical NHEJ (156, 160). Importantly, Polμ is also important for V(D)J recombination (161).

Polν remains one of the least studied members of the human polymerase repertoire. It was initially characterized as a polymerase capable of bypassing certain replication-blocking lesions, including thymine glycol, albeit with low processivity (162, 163).

In cancer, neither Polμ nor Polν has been as extensively studied as other polymerase families, but emerging evidence points to their context-dependent roles. Polμ tumor-associated point mutations have been identified that compromise NHEJ fidelity and reduce the efficiency of accurate DSB repair, with more frequent nucleotide incorporation at break sites (164). These findings suggest that somatic mutations in Polμ could contribute to genomic instability by corrupting the accuracy of NHEJ rather than simply altering polymerase expression levels. Because Polμ’s template-independent synthesis can introduce insertions at repair junctions, its dysregulation could contribute to the indel signatures observed in repair-deficient cancers.

Polν-mediated ICL repair positions it as a potential contributor to the response to crosslinking chemotherapeutics. Loss of Polν has been associated with increased sensitivity to mitomycin C in cellular knockdown models, with *in vitro* evidence also supporting bypass of psoralen-derived ICL intermediates (163). Separately, Polν has been shown to interact physically and functionally with FA pathway factors including FANCD2, and its depletion sensitizes cells to crosslinking agents more broadly (56). Together, these findings suggest that tumors retaining Polν function may have a selective advantage under genotoxic therapy employing crosslinking agents. HPV-associated upregulation of FANCD2 may drive Polν-mediated activity (99, 100).

In cervical cancer, the specific contributions of Polμ and Polν remain largely unexplored, but several lines of reasoning make them plausible participants in HPV-associated tumorigenesis. HPV oncogenes E6 and E7 inactivate p53 and Rb, respectively, driving aberrant cell-cycle entry and replication stress that generates DSBs. Polμ, as a core NHEJ gap-filling polymerase, could be critical for managing these breaks, and any upregulation or mutation of Polμ might alter the fidelity of repair in ways that promote mutagenesis and clonal evolution. One study indicated that suppression of Polμ results in decreased HPV episome stability (96). Similarly, HPV genome integration into host DNA, a process involving strand breakage and often accompanied by complex rearrangements, could invoke NHEJ-dependent repair in which Polμ participates.

For Polν, the chronic replication stress and DNA damage induced by HPV oncoproteins may generate replication fork-associated lesions and crosslink-like intermediates where Polν bypass activity becomes relevant. Although direct evidence strongly linking either polymerase to papillomavirus biology is currently lacking, the dependence of HPV-positive cells on error-prone repair pathways to tolerate persistent genomic damage makes Polμ and Polν attractive candidates for future investigation as contributors to the mutational landscape of cervical cancer.

## THE HPV-ASSOCIATED POLYMERASE MILIEU: NEW FRONTIERS IN DISCOVERY

Only a handful of laboratories actively study the mechanisms of papillomavirus and polymerase engagement across the viral life cycle, which is a striking deficit given that papillomaviruses were first identified nearly a century ago and have been foundational to our understanding of oncogenic transformation. Much remains unknown. Clarifying how low-risk PVs across species induce benign hyperproliferation while high-risk types drive malignancy is not basic science; it is essential for developing new therapeutic strategies. This urgency is compounded by the continued identification of novel PV types, including viruses like equine EcPV2, some of which harbor oncogenic potential that has yet to be characterized (165).

Central to these open questions is the engagement of TLS polymerases during PV replication, a topic that remains poorly understood in any model system. Historically, viruses have served as powerful tools for dissecting fundamental molecular mechanisms, from RNA splicing to the core enzymology of DNA replication, and PV systems are well poised to play a similar role in illuminating TLS biology. Regulatory paradigms for many specialized polymerases have begun to emerge, but the intricacies are far from resolved. PRIMPOL, for instance, appears to be acutely regulated at the protein level by CHK1 and PLK1 while also being transcriptionally upregulated by ATR activation, illustrating the multilayered control these enzymes are subject to (59, 152, 153). A major experimental challenge is that systematic suppression of individual polymerases, whether by siRNA or CRISPRi, can be difficult to interpret in some circumstances due to functional redundancy, demanding that virologists adopt pragmatic and creative approaches to dissect polymerase contributions to PV replication.

Among the more promising of these approaches is iPOND (isolation of proteins on nascent DNA), which enables biochemical interrogation of replisome composition at active replication forks, discussed in further depth in Collins et al. By coupling iPOND with quantitative mass spectrometry, it becomes possible to define the polymerase milieu engaged during PV genome amplification, distinguishing, for example, between processive replicative polymerases and TLS polymerases recruited in response to replication stress or template lesions. Adapted to the PV life cycle, iPOND-based strategies could begin to reveal whether specific polymerase switching events coincide with the transition from maintenance replication in basal cells to viral amplification in differentiating epithelia, a mechanistic space that remains largely unexplored. One caveat, however, is the inability to specifically investigate the HPV-associated replisome. Future technical development to allow for this, or other strategies, is critical to solve this problem.

Complementing these proteomic approaches, advances in long-read sequencing technologies are beginning to offer the fidelity necessary to interrogate PV replication intermediates with real resolution. Unlike short-read approaches, long-read sequencing can capture full-length viral genomes in single reads, preserving structural information about replication intermediates, recombination junctions, and the distribution of polymerase-associated error signatures across the viral genome, as well as incorporation of nucleoside analogs (166). As error rates on these platforms continue to decline, it will become increasingly feasible to map the mutational fingerprints of individual TLS polymerases, such as the characteristic base substitution spectra of Polη, Polκ, or REV1, directly onto nascent PV genomes at the single-molecule level. This would provide a functional readout of which polymerases are active at specific genomic loci and during specific stages of the viral life cycle. Together, iPOND and long-read sequencing represent a convergent methodological framework that has the potential to meaningfully advance our understanding of the polymerase dynamics that underpin PV replication, persistence, and ultimately, oncogenesis.

Adding a structural dimension to these efforts, AlphaFold and related deep learning approaches are making it increasingly possible to model how viral replication proteins like E1 and E2 might interface with TLS polymerases or accessory factors at the fork, generating testable hypotheses about recruitment mechanisms that would be difficult to arrive at through sequence analysis alone (167). When iPOND-identified candidates are mapped onto predicted structural complexes, one can begin to ask whether a given polymerase is a direct binding partner of a viral protein or is instead recruited indirectly through adaptor interactions. As these tools continue to improve, particularly in their handling of transient and context-dependent complexes, this structural layer will become an increasingly valuable complement to the biochemical and genomic approaches described above. Together, iPOND, long-read sequencing, and machine-learning-driven structural prediction represent a convergent toolkit that has the potential to meaningfully advance our understanding of the polymerase dynamics that underpin PV replication, persistence, and ultimately, oncogenesis.

## CONCLUSION

HPVs are elegant, small, and minimal dsDNA elements that not only use the host replication stress response to their own advantage but also dysregulate these factors toward productive replication. They do so by activating host stress and DNA damage responses, which are required for viral fitness. Despite their outward simple appearances, studies indicate that PVs vastly rewire host repair and replication, including dysregulation of at least one TLS polymerase, Polη (60–62). HPVs also inform us how cancers arise.

PVs are suggested to employ high-fidelity polymerases for replication, despite substantial evidence that replication switches modes between viral maintenance and differentiation-dependent amplification. *In vitro* evidence supports at least two high-fidelity host polymerases (Polε and Polα) either interacting and/or their activity being potentiated by HPV E1. Despite this evidence, few studies have been performed across the PV life cycle, investigating longitudinally when these polymerases engage and which are required. The switch to a recombination-based mode of replication is particularly interesting due to its complexity in initiation, homology search, strand invasion, replication, and resolution of all intermediates. Indeed, high-fidelity polymerases can be important in this process generally, but TLS polymerases can also extend D-loops formed during RAD51-mediated strand exchange or BIR, all proposed as models for the switch to differentiation-dependent amplification (27, 168).

The path from Shope’s cottontail rabbit tumors to our current mechanistic understanding of PV-driven replication stress spans nearly a century, yet fundamental questions remain unresolved. Which polymerases are engaged at each stage of the viral life cycle? How does the switch to recombination-based amplification partition fidelity and damage tolerance between high-fidelity and TLS enzymes and does it? Critically, how does viral-driven dysregulation of the host replication stress response contribute to the genomic instability that underlies HPV-associated malignancy? Answering these questions will require longitudinal, stage-specific interrogation of polymerase engagement across the full PV life cycle, an area ripe for future investigation. Shope could not have imagined that the filterable agent he recovered from a Kansas cottontail would one day illuminate the intersection of viral replication, DNA damage tolerance, and cancer biology; we are only beginning to appreciate the depth of that intersection (169).
